# The Thermoregulatory Effect of Immediate Skin-to-Skin Contact for Preterm Infants: A Systematic Review and Meta-Analysis With Trial Sequential Analysis

**DOI:** 10.7759/cureus.90454

**Published:** 2025-08-19

**Authors:** Wardah Albzea, Hanaa F Alrashidi, Shahad Alsanea, Dhouha Alrakaf, Danah AlOmani, Fatemah Alazemi

**Affiliations:** 1 Department of Internal Medicine, Faculty of Medicine, Alexandria University, Alexandria, EGY; 2 Department of Obstetrics and Gynecology, Kuwait Institute for Medical Specializations, Kuwait City, KWT; 3 Department of Obstetrics and Gynecology, ⁠Farwaniyah Hospital, Kuwait City, KWT; 4 Department of Obstetrics and Gynecology, ⁠Maternity Hospital, Kuwait City, KWT; 5 Department of Obstetrics and Gynecology, Jaber Al-Ahmed Hospital, Kuwait City, KWT; 6 Department of Family and Community Medicine, Al-Rabya Health Care Centre, Kuwait City, KWT

**Keywords:** icu, kangaroo care, neonatal, nicu, systematic review

## Abstract

Neonatal thermoregulation poses a considerable challenge, particularly in premature or low-birth-weight infants. Skin-to-skin contact (SSC) is now a recommended strategy to maintain thermoregulation in term infants. Current evidence shows promising results for the application of immediate SSC in preterm infants. A systematic review and meta-analysis synthesizing evidence from randomized controlled trials (RCTs) obtained from PubMed, Google Scholar, CENTRAL, Scopus, and Web of Science until May 2025. Using Stata MP v. 17 (StataCorp LLC, College Station, TX, US), we pooled dichotomous outcomes and continuous outcomes, using relative risk (RR) and standardized mean difference, respectively, along with a 95% confidence interval (CI). Five trials and 401 patients were included in our analysis. Temperature was significantly lower in the SSC group after 60 minutes (MD: -0.21, 95% CI (-0.30, -0.12), P < 0.001). However, there was no significant difference between the two groups regarding hypothermia (RR: 1.23, 95% CI (0.71, 2.16), P = 0.46) and hyperthermia (RR: 0.73, 95% CI (0.52, 1.03), P = 0.07). Also, there was no significant difference between both groups regarding hypoglycemia (RR: 3.15, 95% CI (0.34, 29.37), P = 0.31), stability of the cardiorespiratory system in preterm infants (SCRIP) score (MD: 0.22, 95% CI (-0.10, 0.54), P = 0.18), breathing support (RR: 0.92, 95% CI (0.71, 1.22), P = 0.55), and surfactant administration (RR: 0.77, 95% CI (0.40, 1.45), P = 0.41). Immediate SSC for preterm infants showed a slight decrease in temperature after 60 minutes, showing promising tolerability. Still, despite uncertain evidence, this effect did not impact any other clinical outcome, including hypothermia, hyperthermia, hypoglycemia, SCRIP score, breathing support, or surfactant administration.

## Introduction and background

Thermoregulation presents a significant challenge for neonates, particularly those born prematurely or with low birth weight [1]. Heat is dissipated through convection, radiation, conduction, and evaporation. The high surface area to mass ratio, limited subcutaneous fat, rapid respiration, and underdeveloped metabolism of premature infants put them at risk for hypothermia [2]. Besides internal factors, thermoregulation in newborns is influenced by environmental conditions, such as humidity and temperature, as well as the frequency of procedures that expose them to ambient temperatures [2]. Standard newborn care employs several methods to prevent heat loss, including wrapping the baby in fabric or plastic, providing heated, humidified air through respiratory support tubes and incubators, and utilizing radiant warmers [3]. A body temperature of 36.5-37.4 °C is considered thermostable, with temperatures above this considered hyperthermia [2]. Hypothermia is categorized as mild (36.0-­36.4 °C), moderate (32.0-­35.9 °C), and severe (<32 °C) [4].

Cold stress's interference with the physiological adaptation to extrauterine circulation contributes to the increased morbidity and mortality associated with early-onset hypothermia [1,4,5]. In high-resource settings, a 1 °C decrease in temperature below normothermia upon admission to the neonatal intensive care unit (NICU) is associated with an 11% increase in late-onset sepsis and a 28% increase in mortality among preterm infants [6]. Therefore, investigating strategies to mitigate early-onset hypothermia is of the utmost importance. Kangaroo Mother Care (KMC) resulted in a 72% reduction in hypothermia among stabilized low-birthweight infants in low-resource settings, according to a Cochrane review [7]. Skin-to-skin contact (SSC), adapted from the KMC strategy, is now implemented in high-resource settings for infants in intensive care, with caregivers providing both intermittent and continuous sessions [8].

The initial hour postpartum, a critical period of physiological transition from the intrauterine to the extrauterine environment, is frequently referred to as the "golden hour" [9]. Early SSC was reported to improve temperature control in preterm and term infants [10,11]. Additionally, recent trials have demonstrated promising evidence that immediate SSC for preterm infants can enhance thermoregulation and associated clinical outcomes [2,3,12-15]. However, the evidence remains inconclusive due to small sample sizes and inconsistent outcome reporting. Thus, we conducted this systematic review and meta-analysis of randomized controlled trials (RCTs) to investigate the effect of immediate SSC on thermoregulation in preterm infants.

## Review

Methodology

The Preferred Reporting Items for Systematic Reviews and Meta-Analyses (PRISMA) statement and the Cochrane Handbook for Systematic Reviews of Interventions guided the conduction of this systematic review [16,17].

Data Sources and Search Strategy

On May 18, 2025, an electronic search was conducted on the following databases: Web of Science (WOS), PubMed, Embase, Scopus, and CENTRAL. The search strategy included the following search entries: "(skin-to-skin OR "skin to skin" OR "skin to skin contact" OR "Kangaroo care" OR "skin to skin care" OR "Kangaroo Mother Care" OR KMC OR "maternal skin-to-skin contact" OR "tactile stimulation" OR "tactile contact" OR "parent-infant contact" OR "parent-infant bonding") AND (preterm OR premature OR "low birth weight" OR "gestational age < 37 weeks" OR "gestational age 28-32 weeks" OR "gestational age < 28 weeks") AND ("immediate" OR "early" OR "first hour" OR "golden hour" OR "within 24 hours" OR "immediate postnatal" OR "early postnatal")". The detailed search strategy for each database is depicted in the Appendices. 

No restrictions were applied based on language, country of origin, or publication status. To ensure a thorough and comprehensive review, we also manually screened the reference lists of all included studies and searched trial registries, including ClinicalTrials.gov and the World Health Organization International Clinical Trials Registry Platform. Additional sources, such as ResearchGate, were explored for potentially eligible unpublished studies. Where necessary, corresponding authors were contacted to obtain missing data or clarify study details.

Eligibility Criteria

RCTs conducted using the following PICO criteria were included: population (P), preterm neonates born alive before 37 weeks of pregnancy; intervention (I), immediate SSC in the delivery room; control (C), standard care; and outcomes (O): the primary outcome was thermoregulation, including the mean temperature after 60 minutes, the incidence of hypothermia (hypothermia is categorized as mild (36.0-­36.4 °C), moderate (32.0-­35.9 °C), and severe (<32 °C) [4]), and hyperthermia. The secondary outcomes incorporated the incidence of hypoglycemia, surfactant use, any breathing interventions, and the stability of the cardiorespiratory system in premature infants (SCRIP) score. Furthermore, our analysis excluded RCTs involving term neonates, quasi-randomized trials, conference presentations and proceedings, observational studies, in vitro research, and review articles.

Study Selection

Two independent reviewers used Covidence for a comprehensive screening process. Following duplicate removal, a two-step process was implemented--initial title and abstract screening, followed by a full-text review of the remaining articles. Reviewers resolved their disagreements through discussion and consensus.

Data Extraction

A preliminary extraction of eligible publications was performed to develop a Microsoft Excel extraction form. The form was structured into three sections: (1) summary characteristics of the included trials (study ID, country, study design, sample size, treatment protocols, main inclusion criteria, primary outcome, and follow-up duration); (2) baseline characteristics of the included participants (age, gender, gestational age, birth weight, and cesarean section); and (3) the outcomes sheet (the mean temperature after 60 minutes, hypothermia, hyperthermia, hypoglycemia, surfactant use, any breathing interventions, and the SCRIP score).

The SCRIP score is a validated clinical assessment tool used to evaluate cardiopulmonary function in neonates and infants, particularly in the context of congenital heart disease. It incorporates parameters such as heart rate, respiratory rate, oxygen saturation, and supplemental oxygen requirements, providing an objective measure of physiological stability and guiding perioperative management decisions [13].

Two reviewers independently extracted the data, resolving disagreements through discussion with a senior author. Dichotomous data were documented as event rates, whereas continuous data were summarized using means and standard deviations. Mean and standard deviation were calculated from the median and interquartile range (or range) reported in some included studies using the conversion formulas presented by Wan et al. [18].

Risk of Bias and Certainty of Evidence

The risk of bias in the included studies was assessed using the revised Cochrane Collaboration tool for RCTs (ROB 2) [19]. Two independent reviewers evaluated each study, assessing domains including selection, performance, reporting, and attrition biases, as well as other potential sources of bias. Disagreements were settled via a consensus-building approach. Furthermore, the GRADE (Grading of Recommendations Assessment, Development, and Evaluation) framework was used to assess the certainty of the evidence [20,21]. This evaluation incorporated factors including inconsistencies, imprecision, indirectness, publication bias, and the risk of bias. A thorough analysis of each factor was performed; all decisions were justified and documented. Disagreements in the evaluation process were resolved through collaborative discussion.

Meta-Analysis

Data analysis was conducted with Stata MP version 17 (StataCorp LLC, College Station, TX, US). Dichotomous outcomes were analyzed using the risk ratio (RR), whereas continuous outcomes were analyzed using the mean difference (MD); both are presented with 95% confidence intervals (CI). Standardized mean difference (SMD) was employed for pain assessment due to the varying scores across the included trials. The fixed-effect model was the primary approach; however, a random-effects model was adopted in the presence of significant heterogeneity. Heterogeneity among the included studies was assessed using the chi-squared test and the I-squared statistic (I²), with a p-value of <0.1 for the chi-square test and an I² value of 50% or higher indicating significant heterogeneity. Publication bias was not assessed, as all evaluated outcomes included fewer than 10 RCTs [22]. Finally, trial sequential analysis (TSA) was conducted to assess the robustness and conclusiveness of the meta-analytic results. The TSA considers the size of the information and the cumulative z-curve to determine the sufficiency and robustness of the available evidence. Boundary controls were established to manage the risks associated with Type I and Type II errors. TSA was conducted using the Trial Sequential Analysis software [23].

Results

Search Results and Study Selection

**Figure 1:**
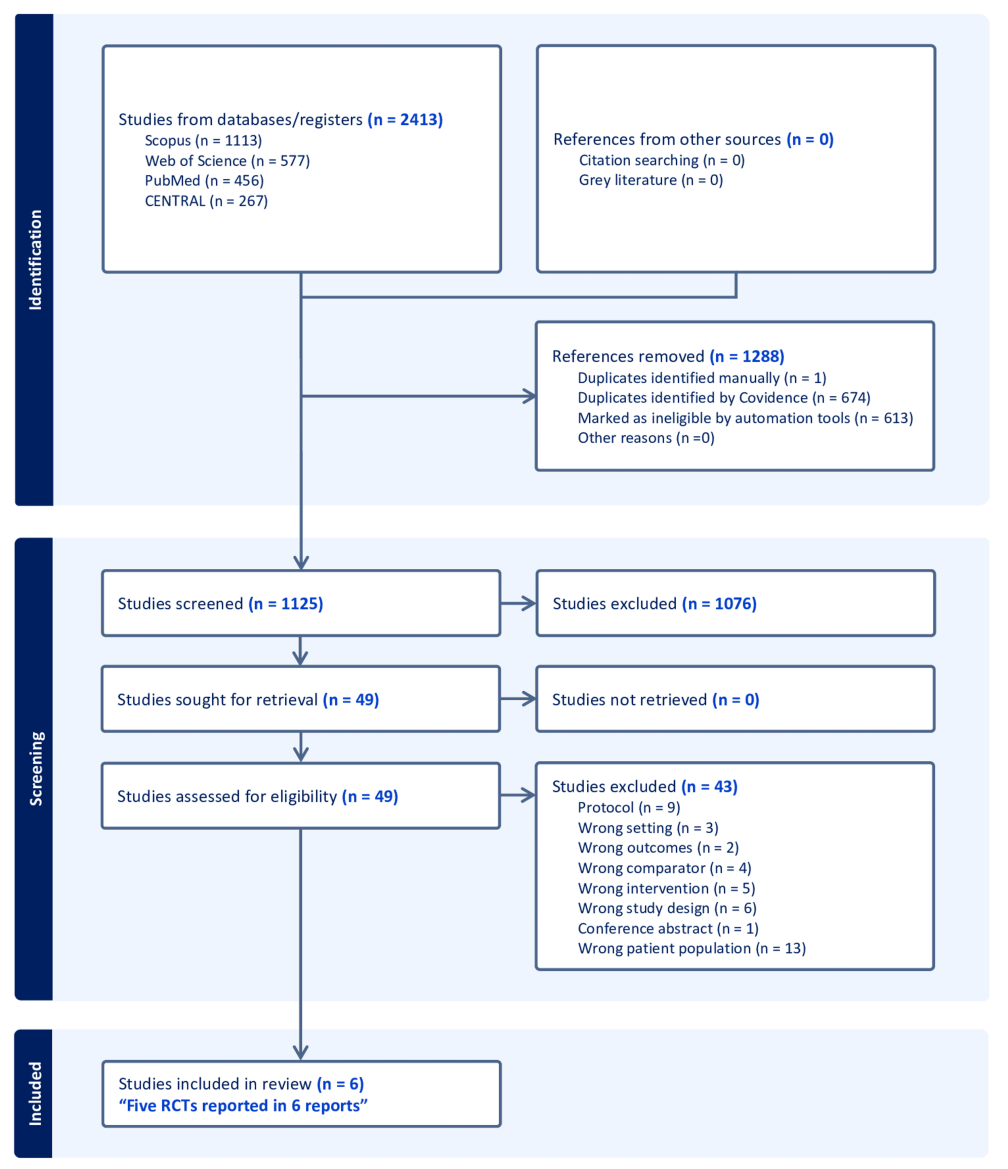
Preferred Reporting Items for Systematic Reviews and Meta-Analyses flow chart of the screening process

Following a search of databases, 2,413 records were identified, and Covidence automatically removed 1,288 irrelevant records, leaving 1,125 records to be screened. Following title and abstract screening, 1,076 studies failed to meet the inclusion criteria and were excluded, leaving 49 full-text articles for further assessment. Forty-three studies were excluded, leaving five RCTs reported in six reports to be included in the qualitative and quantitative analysis (Figure 1) [2,3,12-15].

Characteristics of Included RCTs

Five trials and 401 patients were included in our assessment [2,3,12-15]. The IPISTOSS (Immediate Parent-Infant Skin-To-Skin Study) trial was reported in two studies: a thermoregulation report and a cardiorespiratory stabilization report [2,3]. All trials were conducted in high-income settings [2,3,12,14,15,24], except Singh et al., which was conducted in India (a lower-middle income country) [13]. Further details about the included trials are available in Table 1. Male infants constituted 62.9% of the skin-to-skin group and 56.0% of the control group. More information about the included population is available in Table 2.

**Table 1:** Summary characteristics of the included RCTs RCT = randomized controlled trial, GA = gestational age, SSC = skin-to-skin contact, SCRIP = stability of the cardiorespiratory system in premature infants, NA = not available, NICU = Neonatal Intensive Care Unit

Study ID	Study Design	Country (Clinical Setting)	Total Participants	SSC Details (Place/Delay/Duration)	Control	Main Inclusion Criteria	Primary Outcome
Kristoffersen et al. 2023 [12]	RCT	Norway (St. Olav's hospital, Drammen hospital, and Sørlandet hospital)	N = 108	SSC position on the mother’s chest (Delivery room/just after stabilization/ 2 hours)	Transfer to the NICU incubator	Preterm infants born at GA 280–316 weeks with birth weight >1000 g.	Neurodevelopmental outcome at 2 years corrected age
						At one of the participating hospitals, twins were included and randomized to the same intervention, and at the other two hospitals, only singletons were included	
Linnér et al. 2019 [15]	RCT	Sweden (Danderyd hospital)	N = 55	SSC put on the bare chest of the parent or partner while being dried (Delivery room/immediate/NA)	Transfer to the NICU incubator	Singleton birth within GA 28 + 0‐33 + 6 weeks, roughly corresponding to birthweights 1000‐2500 g	Temperature on admission to the neonatal unit
IPISTOSS Trial [2,3]	RCT	Sweden and Norway (Huddinge and Danderyd at Karolinska University Hospital and at Stavanger University Hospital in Stavanger)	N = 91	SSC with a caregiver started as soon as possible after birth (Delivery room/ immediate/ 6 hours)	Omnibed incubator	Infants born at 28+0 to 32+6 GA regardless of the mode of birth and a parent or surrogate caregiver prepared to start SSC in the first postnatal hour	SCRIP score
Singh et al. 2023 [13]	RCT	India (NA)	N = 100	The SSC group was placed on the mother’s abdomen and dried (Delivery room/ immediate/1 hour)	Radiant warmer	Neonates born at 33/7 to 36/7 weeks of gestation by vaginal delivery were eligible for enrolment in the study if they were breathing spontaneously birth	SCRIP score
Walsh et al. 2020 [14]	RCT	USA (University Hospitals Cleveland Medical Center)	N = 47	SSC position on the mother’s chest (Delivery room/ immediate/ NA)	Radiant warmer	Singletons born at 35 0/7 to 36 6/7 weeks GA	

**Table 2:** Baseline characteristics of the participants N = number of patients, NA = not available, SD = standard deviation, IQR = interquartile range

Study ID	Number of Patients in Each Group		Gender (Male), N (%)		Gestational age (week), Mean (SD)		Birth weight (g), Mean (SD)		APGAR Score at 5 minutes, Median (IQR)		Cesarean Section, N (%)	
	Skin-to-skin Care	Control	Skin-to-skin Care	Control	Skin-to-skin Care	Control	Skin-to-skin Care	Control	Skin-to-skin Care	Control	Skin-to-skin Care	Control
													Kristoffersen et al. 2023 [12]	51	57	33 (65)	35 (61)	30.3 (1.1)	30.3 (1.2)	1436 (266)	1438 (257)	9 (8-10)	9 (8-10)	30 (59)	32 (56)
Linnér et al. 2019 [15]	26	29	17 (65)	16 (55)	31 + 6 ± 10	32 + 0 ± 10	1646 ± 439	1864 ± 439	NA	NA	16 (62)	13 (45)
IPISTOSS Trial [2,3]	46	45	33 (72)	18 (40)	31+2	31+0	1571 ± 395	1494 ± 400	9 (7–9)	9 (8–10)	22 (63)	25 (77)
Singh et al. 2023 [13]	50	50	28 (56)	31 (62)	34.8 (1.2)	34.7 (1.2)	2340 (449)	2283 (453)	9 (9–9)	9 (9–9)	NA	NA
Walsh et al. 2020 [14]	21	26	11 (52.4)	16 (61.5)	NA	NA	NA	NA	NA	NA	NA	NA

Risk of Bias and Certainty of Evidence

**Figure 2:**
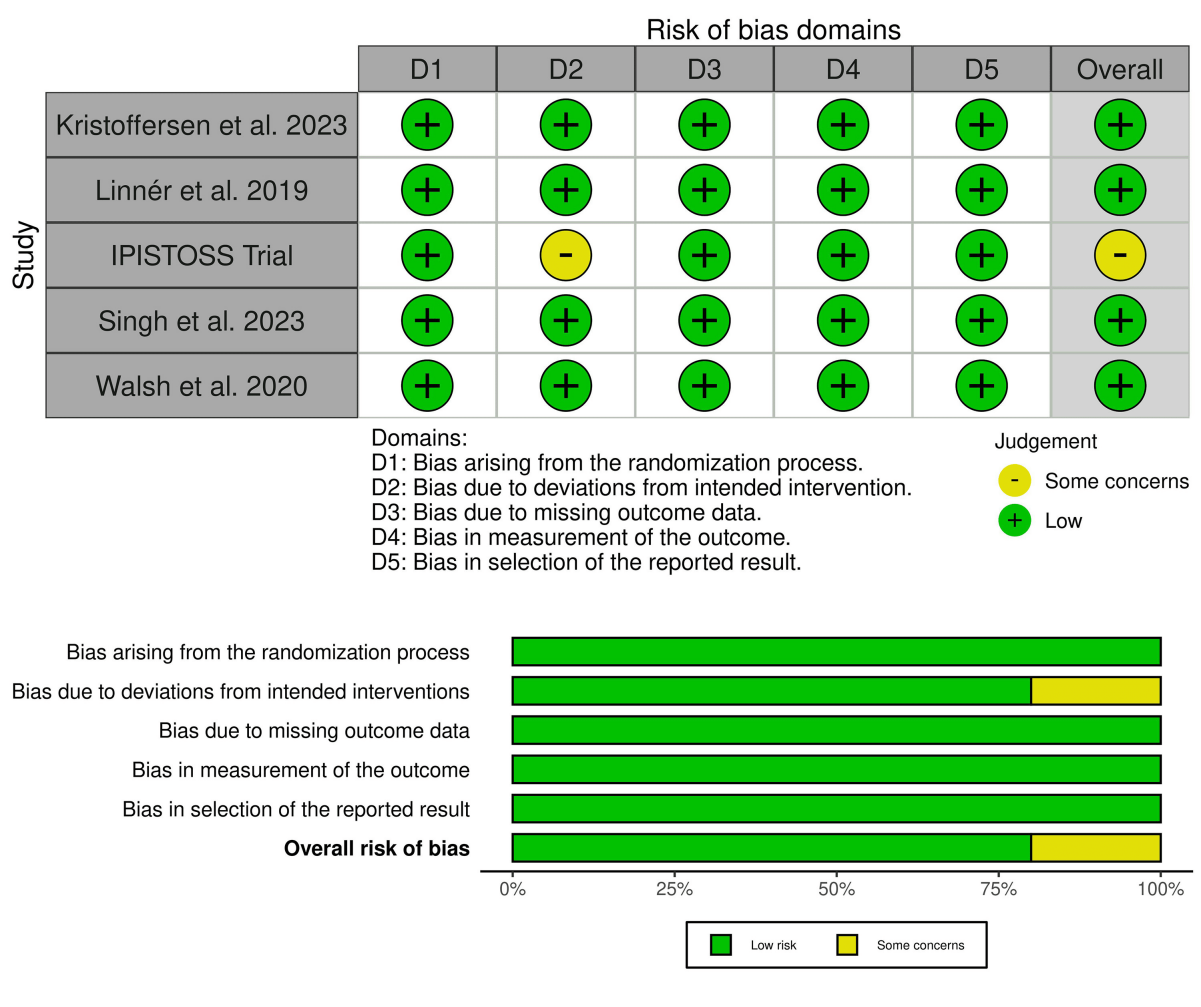
Quality assessment of risk of bias in the included trials The upper panel presents a schematic representation of risks (low = red, some concerns = yellow, and high = red) for specific types of biases in the studies in the review. The lower panel presents risks (low = red, some concerns = yellow, and high = red) for the subtypes of biases of the combination of studies included in this review. [2,3,12-15]

All included trials showed a low risk of bias, except for the IPISTOSS trial, which raised some concerns about bias (Figure 2). The IPISTOSS trial raised concerns about performance bias, as the neonatologist in charge decided whether to initiate SSC immediately or first assess the newborn infant under radiant heat during resuscitation [2,3]. Certainty of evidence is illustrated in Table 3.

**Table 3:** GRADE evidence profile RCTs = randomized controlled trials, CI = confidence interval, MD = mean difference, RR = risk ratio, SCRIP = stability of the cardiorespiratory system in premature infants Explanations: a. A wide confidence interval that does not exclude the appreciable harm/benefit, with a low number of events. b. The IPISTOSS trial raised concerns about bias, with more than 50% of the pooled analysis weight. c. A wide confidence interval that does not exclude the appreciable harm/benefit, with a low number of participants.

Certainty assessment							Summary of findings				
Participants (studies) Follow-up	Risk of bias	Inconsistency	Indirectness	Imprecision	Publication bias	Overall certainty of evidence	Study event rates (%)		Relative effect (95% CI)	Anticipated absolute effects	
							With (Control)	With (Skin-to-skin care)		Risk with (Control)	Risk difference with (Skin-to-skin care)
Axillary Temperature After 60 Minutes											
401 (5 RCTs)	not serious	not serious	not serious	not serious	none	⨁⨁⨁⨁ High	207	194	-	207	MD 0.21 °C lower (0.3 lower to 0.12 lower)
Hypothermia											
354 (4 RCTs)	not serious	not serious	not serious	very serious^a^	none	⨁⨁◯◯ Low^a^	18/181 (9.9%)	23/173 (13.3%)	RR 1.23 (0.71 to 2.16)	18/181 (9.9%)	23 more per 1,000 (from 29 fewer to 115 more)
Hyperthermia											
199 (2 RCTs)	serious^b^	not serious	not serious	very serious^a^	none	⨁◯◯◯ Very low^a,b^	59/102 (57.8%)	36/97 (37.1%)	RR 0.73 (0.52 to 1.03)	59/102 (57.8%)	156 fewer per 1,000 (from 278 fewer to 17 more)
Hypoglycemia											
208 (2 RCTs)	not serious	not serious	not serious	extremely serious^a^	none	⨁◯◯◯ Very low^a^	0/107 (0.0%)	2/101 (2.0%)	RR 3.15 (0.34 to 29.37)	0/107 (0.0%)	0 fewer per 1,000 (from 0 fewer to 0 fewer)
SCRIP Score											
191 (2 RCTs)	not serious	not serious	not serious	very serious^c^	none	⨁⨁◯◯ Low^c^	95	96	-	95	MD 0.22 higher (0.1 lower to 0.54 higher)
Breathing Support											
208 (2 RCTs)	not serious	not serious	not serious	very serious^a^	none	⨁⨁◯◯ Low^a^	73/107 (68.2%)	61/101 (60.4%)	RR 0.92 (0.71 to 1.20)	73/107 (68.2%)	55 fewer per 1,000 (from 198 fewer to 136 more)
Surfactant Use											
158 (2 RCTs)	not serious	not serious	not serious	very serious^a^	none	⨁⨁◯◯ Low^a^	20/81 (24.7%)	13/77 (16.9%)	RR 0.77 (0.40 to 1.45)	20/81 (24.7%)	57 fewer per 1,000 (from 148 fewer to 111 more)

Primary Outcomes: Thermoregulation

**Figure 3:**
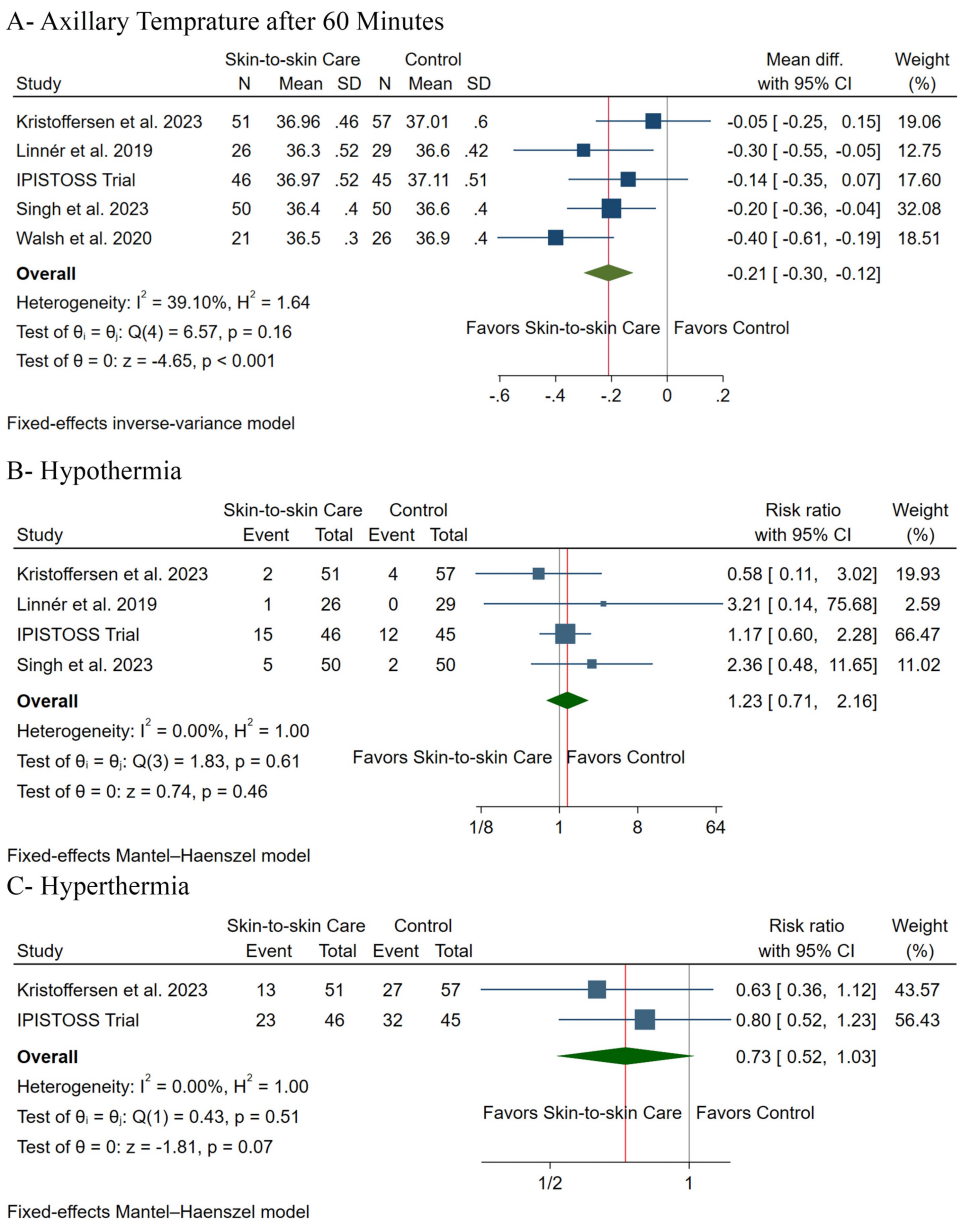
Forest plot of the primary outcomes of thermoregulation [2,3,12-15] N = number of patients, SD = standard deviation, CI = confidence interval

Temperature was significantly lower in the SSC group after 60 minutes (MD: -0.21, 95% CI (-0.30, -0.12), P< 0.001) (Figure 3A). However, there was no significant difference between both groups regarding the incidence of hypothermia (RR: 1.23, 95% CI (0.71, 2.16), P= 0.46) (Figure 3B) and hyperthermia (RR: 0.73, 95% CI (0.52, 1.03), P= 0.07) (Figure 3C). Pooled studies were homogeneous in temperature after 60 minutes (I2 = 39%), hypothermia (I2 = 0%), and hyperthermia (I2 = 0%).

**Figure 4:**
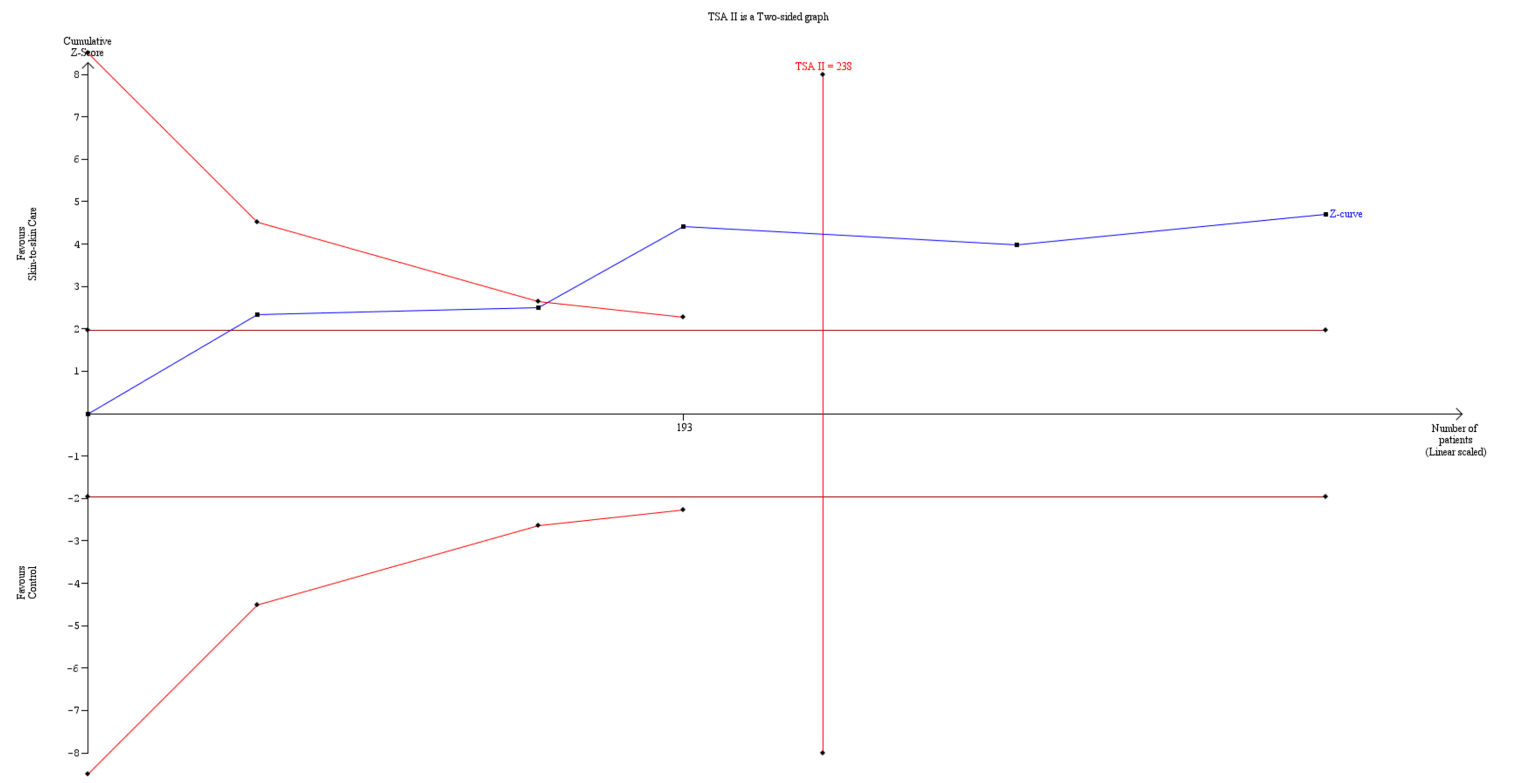
Trial sequential analysis of temperature after 60 minutes TSA = Trial Sequential Analysis

TSA showed that the cumulative Z-curve (blue line) crossed the trial sequential monitoring boundary for benefit (red upper boundary) before reaching the required information size, indicating that the observed effect is statistically significant and unlikely to be due to random error (Figure 4).

Secondary Outcomes

**Figure 5:**
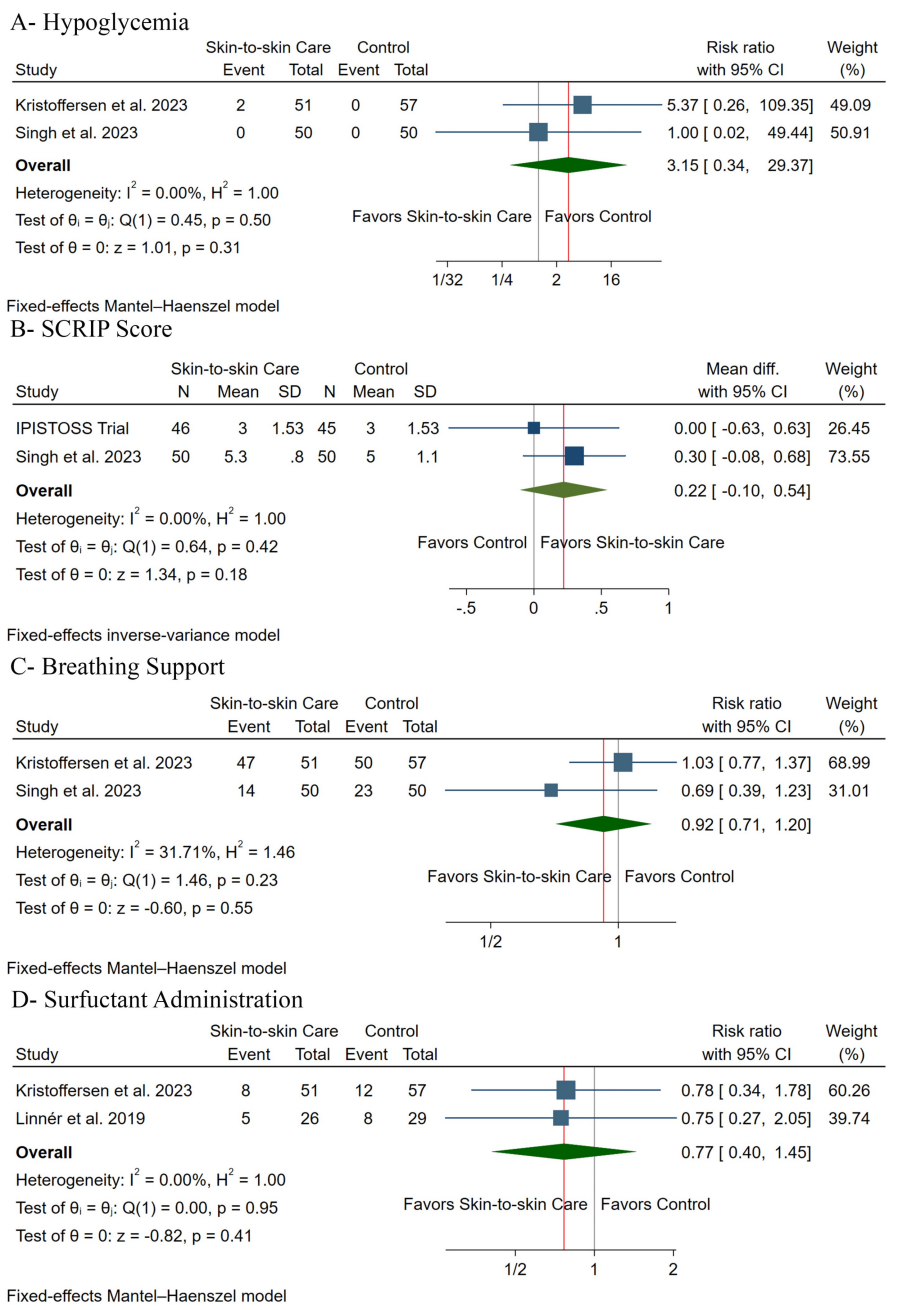
Forest plot of the secondary outcomes [2,3,12-15] N = number of patients, SD = standard deviation, CI = confidence interval

There was no significant difference between both groups regarding the incidence of hypoglycemia (RR: 3.15, 95% CI (0.34, 29.37), P= 0.31) (Figure 5A), SCRIP score (MD: 0.22, 95% CI (-0.10, 0.54), P= 0.18) (Figure 5B), breathing support (RR: 0.92, 95% CI (0.71, 1.22), P= 0.55) (Figure 5C), and surfactant administration (RR: 0.77, 95% CI (0.40, 1.45), P= 0.41) (Figure 5D). Pooled studies were homogeneous in hypoglycemia (I2 = 0%), SCRIP score (I2 = 0%), breathing support (I2 = 32%), and surfactant administration (I2 = 0%).

Discussion

Evidence from five RCTs, which included 401 preterm infants, showed that SSC led to a slight decrease in axillary temperature compared to the control group. Also, there was no significant effect on the incidence of hypothermia, hyperthermia, or hypoglycemia. This aligned with comparable cardio-respiratory function, as assessed by the SCRIP score, and similar management parameters for breathing support and surfactant administration. Finally, TSA for temperature after 60 minutes indicated that the cumulative Z-curve crossed the monitoring boundary for benefit before reaching the required information size, suggesting that the observed effect on mean temperature is statistically significant and robust, unlikely to be due to random error.

Although statistical heterogeneity was not detected in our pooled analyses, potential clinical heterogeneity may still exist. Differences in baseline characteristics (e.g., gestational age, birth weight, comorbidities) and variations in intervention protocols (e.g., delivery method) among the included trials could influence treatment effects. Such variability, while not reflected in statistical measures, may limit the generalizability of our findings and should be considered when interpreting the results.

Multiple clinical guidelines endorsed SSC as a vital strategy for thermoregulation in neonates. The World Health Organization and the American Academy of Pediatrics recommend SSC for preterm neonates [8,25]. Notably, the Canadian Paediatric Society highlights that immediate postpartum SSC facilitates the stabilization of physiological parameters, such as temperature, and promotes enhanced thermoregulation and a modest temperature elevation in premature infants [26]. However, our findings showed that SSC slightly decreased the temperature after 60 minutes.

The interpretation of this finding hinges on reconciling the statistically reduced mean temperature with the insignificant difference observed in hypothermia and hyperthermia rates. This indicates that, although the mean temperature in the SSC group was slightly lower, it largely stayed within the clinically acceptable normothermic range (36.5-37.4 °C). While statistically significant, the mean difference of 0.21 °C may lack sufficient clinical relevance to cause a notable increase in infants experiencing mild hypothermia (defined as a temperature range of 36.0-36.4 °C). The slightly reduced mean temperature may be a positive finding, suggesting that a more stable and appropriate thermoregulation is achieved through maternal contact rather than a detrimental effect, and in contrast to the use of external warming devices. Body temperature is dynamically regulated in all humans, including preterm infants. The mother's body acts as a more responsive and even temperature regulator than a potentially overshooting external device, providing warmth as needed while preventing overheating, which can result in a slightly lower but more stable temperature [25].

Additionally, a pooled analysis showed some variability in temperature after one hour, with a relatively low heterogeneity of 39%. Several factors may contribute to this variability, including the temperature in the delivery room or NICU, specific SSC protocols (e.g., timing of initiation, duration, use of coverings), and the infant's initial condition [27,28]. Notably, the body temperature benefits of SSC are more significant in colder conditions. A cooler initial environment for SSC could result in a slightly lower temperature than a warmed incubator, although it would still be within safe limits [28].

Furthermore, we found no significant difference in the incidence of hypoglycemia between the immediate SSC and control groups. This result is in contrast to a previous systematic review and meta-analysis examining SSC for the prevention of neonatal hypoglycemia, which included 7 RCTs and 922 infants, concluding that SSC may lead to a substantial reduction in the incidence of neonatal hypoglycemia [29]. The inconsistencies observed in the findings are probably a result of the significant imprecision inherent in the current analysis. This inaccuracy stems from the inclusion of only two RCTs for this outcome, far fewer than the seven included in the previous meta-analysis [29]. Therefore, the non-significant finding should be interpreted as a lack of sufficient evidence to conclude definitively, not as proof of no effect.

Moreover, we found no significant differences in SCRIP score and breathing support between the immediate SSC and control groups. Although this analysis found no significant effect, existing research generally suggests that SSC has a positive impact on cardiorespiratory stability [26]. SSC also contributes to the stabilization of key physiological functions such as heart rate and oxygen saturation. A previous meta-analysis indicated improved oxygen saturation and heart rate and even suggested a protective effect against apnea of prematurity [28]. Similar to the findings for hypoglycemia, the non-significant results for SCRIP score and breathing support are likely a consequence of the limited number of included studies (only 2 RCTs for each outcome, involving 191-208 participants) and the resulting "very serious imprecision" in the GRADE assessment. Hence, due to insufficient statistical power, this meta-analysis is unlikely to detect clinically meaningful effects on these well-established outcomes.

The review process strictly adhered to PRISMA guidelines and the Cochrane Handbook for Systematic Reviews of Interventions. The study utilizes only RCTs, thus presenting the gold standard in evidence-based research. Additionally, the GRADE framework's integration and TSA application facilitated a systematic and transparent evaluation of evidence certainty for each outcome, thereby indicating the trustworthiness and confidence levels associated with the effect estimates. Still, our review is limited by the following: First, the analysis included only five trials overall; several secondary outcomes were based on a mere two trials. This limitation inherently reduces the statistical power of the meta-analysis, resulting in wide confidence intervals and a GRADE assessment classification of "very serious" or "extremely serious imprecision" for most outcomes. Second, publication bias was not formally assessed for any outcome because all evaluated outcomes included fewer than 10 RCTs. Third, although low statistical heterogeneity (low I²) was demonstrated across most outcomes in the pooled studies, variability persisted in the immediate SSC protocols employed across the included trials. Although the low I² values suggest that the core intervention (immediate SSC) may have a consistent effect, regardless of minor protocol variations or due to an insufficient sample size to detect heterogeneity, these variations hinder the definition of an optimal SSC protocol based solely on this meta-analysis. Also, we did not prospectively register our protocol in PROSPERO, which may raise concerns about selective outcome reporting. To address this, we closely followed established methodological guidelines and strictly adhered to our predefined objectives and inclusion criteria. All reported outcomes were based on extracted data, with no selective additions or omissions. Finally, this meta-analysis focused on a specific set of immediate physiological and clinical outcomes. This review did not assess other substantial benefits associated with SSC, including elevated breastfeeding rates, strengthened parent-infant bonding, potential long-term neurodevelopmental gains, and decreased mortality.

The limitations highlighted in this meta-analysis necessitate further research in several critical areas to enhance the evidentiary basis for immediate skin-to-skin contact in preterm infants. Large, multicenter RCTs are urgently needed, especially to assess secondary outcomes, including hypoglycemia, SCRIP score, respiratory support, and surfactant administration. Sufficient statistical power is required in future studies to identify clinically relevant differences and mitigate the considerable imprecision noted in this meta-analysis. Also, subsequent trials should incorporate pragmatic design, thereby facilitating real-world applicability without compromising methodological rigor. Additional investigation is required to clarify the physiological processes responsible for the marginally reduced mean temperature in the SSC cohort. This could entail sustained temperature monitoring, comprehensive assessment of metabolic rate, and a thorough investigation of maternal thermoregulatory function [28]. Standardization of immediate SSC protocols is a key objective for future studies. The process includes establishing clear definitions for immediate SSC, optimizing contact durations, and ensuring consistent environmental control.

## Conclusions

Immediate SSC for preterm infants showed a slight decrease in temperature after 60 minutes, showing promising tolerability. Still, despite uncertain evidence, this effect did not impact any other clinical outcome, including hypothermia, hyperthermia, hypoglycemia, SCRIP score, breathing support, or surfactant administration. Therefore, future large-scale pragmatic RCTs remain warranted to investigate the effect of immediate SSC on clinical and management outcomes.

## Appendices

**Table 4:** Search strategy

Database	Search Terms	Search Field	Search Results
PubMed	(skin-to-skin OR "skin to skin" OR "skin to skin contact" OR "Kangaroo care" OR "skin to skin care" OR "Kangaroo Mother Care" OR KMC OR "maternal skin-to-skin contact" OR "tactile stimulation" OR "tactile contact" OR "parent-infant contact" OR "parent-infant bonding") AND (preterm OR premature OR "low birth weight" OR "gestational age < 37 weeks" OR "gestational age 28-32 weeks" OR "gestational age < 28 weeks") AND ("immediate" OR "early" OR "first hour" OR "golden hour" OR "within 24 hours" OR "immediate postnatal" OR "early postnatal")	All Fields	456
Cochrane	(skin-to-skin OR "skin to skin" OR "skin to skin contact" OR "Kangaroo care" OR "skin to skin care" OR "Kangaroo Mother Care" OR KMC OR "maternal skin-to-skin contact" OR "tactile stimulation" OR "tactile contact" OR "parent-infant contact" OR "parent-infant bonding") AND (preterm OR premature OR "low birth weight" OR "gestational age < 37 weeks" OR "gestational age 28-32 weeks" OR "gestational age < 28 weeks") AND ("immediate" OR "early" OR "first hour" OR "golden hour" OR "within 24 hours" OR "immediate postnatal" OR "early postnatal")	All Text	267
WOS	(skin-to-skin OR "skin to skin" OR "skin to skin contact" OR "Kangaroo care" OR "skin to skin care" OR "Kangaroo Mother Care" OR KMC OR "maternal skin-to-skin contact" OR "tactile stimulation" OR "tactile contact" OR "parent-infant contact" OR "parent-infant bonding") AND (preterm OR premature OR "low birth weight" OR "gestational age < 37 weeks" OR "gestational age 28-32 weeks" OR "gestational age < 28 weeks") AND ("immediate" OR "early" OR "first hour" OR "golden hour" OR "within 24 hours" OR "immediate postnatal" OR "early postnatal")	All Fields	577
SCOPUS	TITLE ( skin-to-skin OR "skin to skin" OR "skin to skin contact" OR "Kangaroo care" OR "skin to skin care" OR "Kangaroo Mother Care" OR kmc OR "maternal skin-to-skin contact" OR "tactile stimulation" OR "tactile contact" OR "parent-infant contact" OR "parent-infant bonding" ) AND ( preterm OR premature OR "low birth weight" OR "gestational age < 37 weeks" OR "gestational age 28-32 weeks" OR "gestational age < 28 weeks" ) AND ( "immediate" OR "early" OR "first hour" OR "golden hour" OR "within 24 hours" OR "immediate postnatal" OR "early postnatal" )	Title	1,113
